# Berry Dietary Interventions in Metabolic Syndrome: New Insights

**DOI:** 10.3390/nu15081906

**Published:** 2023-04-14

**Authors:** Samuele Venturi, Mirko Marino, Iolanda Cioffi, Daniela Martini, Cristian Del Bo’, Simone Perna, Patrizia Riso, Dorothy Klimis-Zacas, Marisa Porrini

**Affiliations:** 1 Department of Food, Environmental and Nutritional Sciences, Università degli Studi di Milano, 20133 Milan, Italy; samuele.venturi@unimi.it (S.V.); mirko.marino@unimi.it (M.M.); iolanda.cioffi@unimi.it (I.C.); daniela.martini@unimi.it (D.M.); simone.perna@unimi.it (S.P.); patrizia.riso@unimi.it (P.R.); marisa.porrini@unimi.it (M.P.); 2 School of Food and Agriculture, University of Maine, Orono, ME 04469, USA; dorothea@maine.edu

**Keywords:** blueberry, raspberry, strawberry, cranberry, humans, dietary intervention, metabolic syndrome

## Abstract

Metabolic Syndrome (MetS) is characterized by a group of dysmetabolic conditions, including abdominal obesity, dyslipidemia, glucose intolerance and/or insulin resistance, and hypertension. Generally, MetS is accompanied by an exacerbation of oxidative stress, inflammation, and vascular dysfunction. Increasing evidence suggests that berries and berry bioactives could play a potential role in the prevention and mitigation of the risk factors associated with MetS. The present systematic review summarizes the more recently available evidence deriving from human intervention studies investigating the effect of berries in subjects with at least three out of five MetS parameters. The PubMed, Scopus, and Embase databases were systematically searched from January 2010 until December 2022. A total of 17 human intervention trials met the inclusion criteria. Most of them were focused on blueberry (n = 6), cranberry (n = 3), and chokeberry (n = 3), while very few or none were available for the other berries. If considering MetS features, the main positive effects were related to lipid profile (low and high-density lipoproteins, cholesterol, and triglycerides) following blueberries and chokeberries, while conflicting results were documented for anthropometric parameters, blood pressure, and fasting blood glucose levels. Other markers analyzed within the studies included vascular function, oxidative stress, and inflammation. Here, the main positive effects were related to inflammation with a reduction in interleukin 6 and tumor necrosis factor-alpha following the intake of different berries. In conclusion, although limited, the evidence seems to support a potential role for berries in the modulation of lipid profile and inflammation in subjects with MetS. Furthermore, high-quality intervention trials are mandatory to demonstrate the role of berries in reducing risk factors for MetS and related conditions. In the future, such a demonstration could bring the adoption of berries as a potential dietary strategy to prevent/counteract MetS and related risk factors.

## 1. Introduction

Metabolic syndrome (MetS) comprises a series of metabolic alterations known to be linked to adverse health outcomes [1,2,3]. Its definition was harmonized in 2009, starting with the criteria proposed by the National Cholesterol Education Program–Adult Treatment Panel III (NCEP-ATP III), which defines MetS as the condition characterized by at least three out of five risk factors: central obesity (waist circumference (WC) ≥102 cm in males, ≥88 cm in females); high triglycerides (TG) (≥150 mg/dL); low HDL cholesterol (≤40 mg/dL in males, ≤50 mg/dL in females); hypertension (≥130/85 mmHg) and raised fasting glucose (≥100 mg/L). Regarding WC, there are different definitions based on country and population characteristics. Also, the use of hypoglycaemic, antihypertensive, and hypolipidemic drugs are considered alternative indicators for the clinical diagnosis of MetS [4]. Since the components of MetS, either individually or in combination, are crucial risk factors for developing cardiovascular diseases (CVDs) and Type 2 Diabetes (T2D) [5], the implications for future healthcare costs and management appear utterly relevant and challenging. In this regard, in recent years, MetS has become much more common in the United States of America (USA) (over 30% adults) [6,7] and Europe (over 24% adults) [1,8], but it is also increasing globally. Many of the conditions that may contribute to MetS development can be addressed by modifying lifestyles, such as physical exercise and diet. Diet modification represents a pivotal strategy for CVD prevention and particularly since increasing evidence has reported a strong inverse association between CVD risks, MetS, and the consumption of plant foods and plant-based bioactives [9,10,11,12].

Berries (e.g., blueberries, bilberries, cranberries, raspberries, and strawberries) have been recently studied for their capacity to prevent MetS [3,13,14,15]. In fact, berries contain a wide range of nutrients and bioactive compounds such as vitamins (e.g., vitamin C and folic acid), minerals (e.g., potassium, manganese), fiber, but above all (poly)phenols (flavonoids, phenolic acids, condensed and hydrolyzable tannins, stilbenoids, and lignans) [16,17]. Among (poly)phenols, anthocyanins (ACNs) constitute the largest proportion of bioactive polyphenols in berries (range content from 100 to 200 mg per 100 g edible portions) [16]. These components are proposed to be the major compounds responsible for the physiological improvements from berry consumption, and for this reason, they have been extensively studied for their protective effects against CVD. Firm epidemiological evidence has shown that the consumption of berries has been inversely associated with the risk/incidence of hyperlipidemia, insulin resistance, diabetes, and CVD [18,19,20]. Cassidy et al. (2013) found a trend toward reduction in myocardial infarction risk (−34%) in subjects consuming greater than three portions per week of strawberries and blueberries compared to subjects consuming berries less than once a month [18]. In another cross-sectional study, higher habitual ACN intakes (35 mg ~ half a portion of berries) resulted in an improvement in insulin levels [20]. A recent meta-analysis of prospective studies reported a 9% lower risk of coronary heart disease (CHD) for subjects consuming ACN-rich foods [21]. A meta-analysis of 15 prospective cohort studies suggested that high dietary ACNs (>200 mg/day), including those from berries, were associated with a lower risk of coronary heart disease (CHD), total CVD incidence, and total CVD deaths [22]. However, another meta-analysis showed no significant inverse association with incident cardiovascular outcomes and/or events for berries [23].

Both animal models and cell culture studies have investigated the potential mechanisms by which berries and their bioactives may improve MetS parameters. In particular, cell culture models have shown the capacity of berry components to positively affect numerous transcription factors related to lipid metabolism (i.e., CCAAT/enhancer-binding protein β (C/EBPβ), peroxisome proliferator-activated receptor-γ1), inflammatory response (i.e., nuclear factor kappa B), oxidative stress (i.e., transcription factor nuclear factor erythroid 2-related factor 2) even if the mechanisms are not fully understood yet [24,25,26,27]. Human intervention studies have substantially shown the role of berries and their bioactives in improving vascular function and alleviating arterial stiffness among subjects with high CVD [28]. However, results on the effects of berries on body mass index (BMI), fat deposition, blood pressure, and chronic low-grade inflammation are still contradictory, as emerged by a recent meta-analysis of randomized control trials [22]. Another nebulous aspect regards the role of berries on glucose and insulin metabolism. The results of a systematic review and meta-analysis of human intervention studies did not show a clear effect of berry bioactives on biomarkers of glucose metabolism when compared with the placebo group [29]. Although some analyses showed statistically significant effects, such improvements were too small to be of clinical relevance.

Given this discrepancy, the aim of the present study was to provide a comprehensive overview of the more recent evidence from human intervention studies evaluating the effect of berries on subjects with MetS.

## 2. Materials and Methods

### 2.1. Literature Search

**Figure 1:**
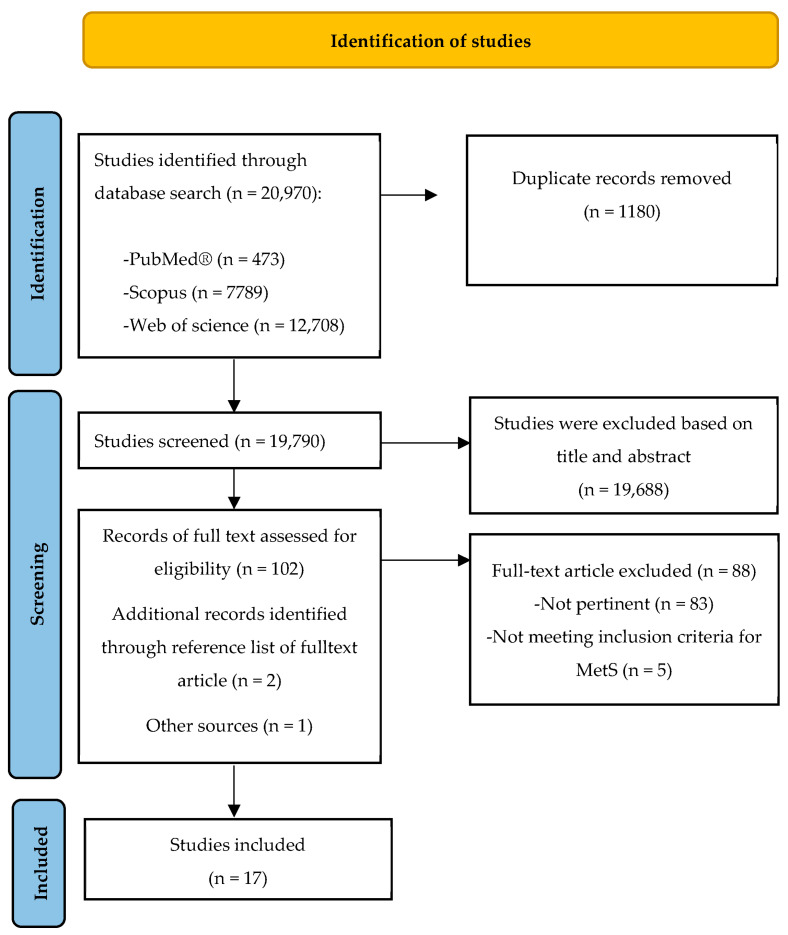
PRISMA flow chart of the systematic review literature search.

The search strategy was carried out on 20 December 2022 and updated on 5 February 2023 by using three different electronic databases: PubMed, Scopus, and Embase. The construction of the search was performed using a combination of the following terms and text words in the databases: “metabolic syndrome” AND “human intervention study” AND “berries” OR “blueberry” OR “cranberry” OR “strawberry” OR “bilberry” OR “raspberry” OR “chokeberry” OR “whortleberry” OR “blackcurrant”. Only papers written in the English language were considered. The PRISMA statement (Preferred Reporting Items for Systematic Reviews and Meta-Analyses) was used for the literature identification process, as illustrated in Figure 1. The review and the protocol were not recorded in any register.

### 2.2. Study Selection

Studies were considered eligible if they: (i) were published between January 2010 and December 2022 (excluding studies performed up to 2010 for the presence of other reviews and meta-analyses on this topic), (ii) were written in English, (iii) consisted of human intervention studies investigating the effects of berry or berry mixtures in the context of MetS, and (iv) involved subjects with a diagnosis of MetS (at least three out of five factors). Studies were excluded if not focused on MetS or if the effect of berries was tested in combination with drugs and/or medications. No further restrictions were applied. In Table 1, the complete list of eligibility criteria, developed by following the Population, Intervention, Comparison, Outcome, and Study design (PICOS) criteria, is reported.

Two reviewers (S.P. and S.V.) independently performed the literature search and assessed studies eligibility based on the title and abstract. Disagreement between reviewers was resolved by a third independent reviewer (M.M).

### 2.3. Data Extraction and Analysis

Two authors (S.V. and C.D.B) independently performed data extraction and analysis of the eligible studies. Disagreement between reviewers was solved by a third independent reviewer (I.C.) to reach a consensus. The following information was collected: (i) the type of berry, (ii) the duration of the treatment, (iii) the study design, (iv) the number and the subjects’ characteristics, (v) the treatment in terms of dose/amount of berry and polyphenols administered, (vi) the main findings, and (vii) first author name and year of publication.

### 2.4. Risk of Bias

Studies were independently analyzed by two authors (I.C. and M.M.). A third author (D.M.) solved potential disagreements. The risk of bias in RCTs was evaluated by using the seven domains developed by the Cochrane Collaboration for RCTs [30]. Non-RCTs were analyzed using the seven domains developed for non-randomized studies of interventions (ROBINS-I) [31]. The domains were judged as follows: “high risk”, “unclear risk”, or “low risk”, following the instructions reported [30].

## 3. Results

### 3.1. Study Selection

A total of 20,970 records were found after consultation with PubMed, Scopus, Embase, and other sources (Figure 1). After the removal of duplicate records (n = 1180) and those excluded based on title and abstract (n = 19,688) or not of interest/pertinent (88 studies), 17 papers that met the criteria were analyzed.

### 3.2. Study Characteristics and Main Findings

The main characteristics of the 17 studies included in the review are summarized in Table 2 [5,32,33,34,35,36,37,38,39,40,41,42,43,44,45,46,47]. Six studies tested the effect of blueberry (BB) [5,32,33,34,35,36], while three studied the effects of cranberry (CB) [38,39,40] and chokeberry (ChB) [43,44,45]. Other berries included: strawberries [46], bilberry [37], and raspberry [41,42]. One study used a mix of berries [47]. Studies were performed in different countries, mainly in the USA (eight studies) and Europe (five studies), followed by Asia (two studies), South America (Brazil, one study), and Canada (one study). Most of the studies were randomized, controlled trials [5,32,33,35,36,37,40,41,46,47]. Two studies were post-prandial interventions [32,34], while the rest of the studies had a duration ranging between four and fourteen weeks (mean of eight weeks) depending on the berry and endpoint considered [5,33,35,36,37,38,39,40,41,42,43,44,45,46,47]. A total of 785 subjects with MetS were included in the studies. The following classifications were used for identifying MetS among patients: NCE-ATP III [48], WHO [49], AHA/NHLBI [50] and the harmonized definition [4]. The largest study recruited 143 subjects [43], while the smallest five subjects [34]. Participants were in the range of 26–70 years, with a high prevalence of subjects in the age range of 45–65 years. The BMI ranged between 25–40 kg/m^2^, with a high prevalence of subjects with a BMI above 30 kg/m^2^. Most of the subjects used drugs/medications for the control of blood cholesterol (e.g., statin) and blood pressure (antihypertensive). In some studies, subjects also consumed supplements (multivitamins).

Berries were administered mainly in the form of beverages, fruit, or extract. For the beverage, the most frequent form was the freeze-dried powder (dose range 13–50 g) resuspended in water (amount range 125–480 mL). The amount ranged from 75 to 350 g for fresh blueberries, 200–300 g for bilberries and raspberries, and up to 500 g for strawberries. The total (poly)phenols provided by berries was in the range of 200–800 mg; however, some studies provided up to 1000 mg [32,33] or 2000 mg of total (poly)phenols [46]. Three studies did not report data in terms of (poly)phenols and/or their subclasses [38,41,42].

Regarding markers, the main parameters related to MetS conditions included: blood pressure, lipid profile, glycemia, and waist circumference. In addition, the following markers were also considered: oxidative stress, inflammation, and vascular function. Some studies also analyzed gut microbiota composition [47], metabolome [47], and markers of immune function [5] (Supplementary Table S1).

### 3.3. Risk of Bias

**Figure 2:**
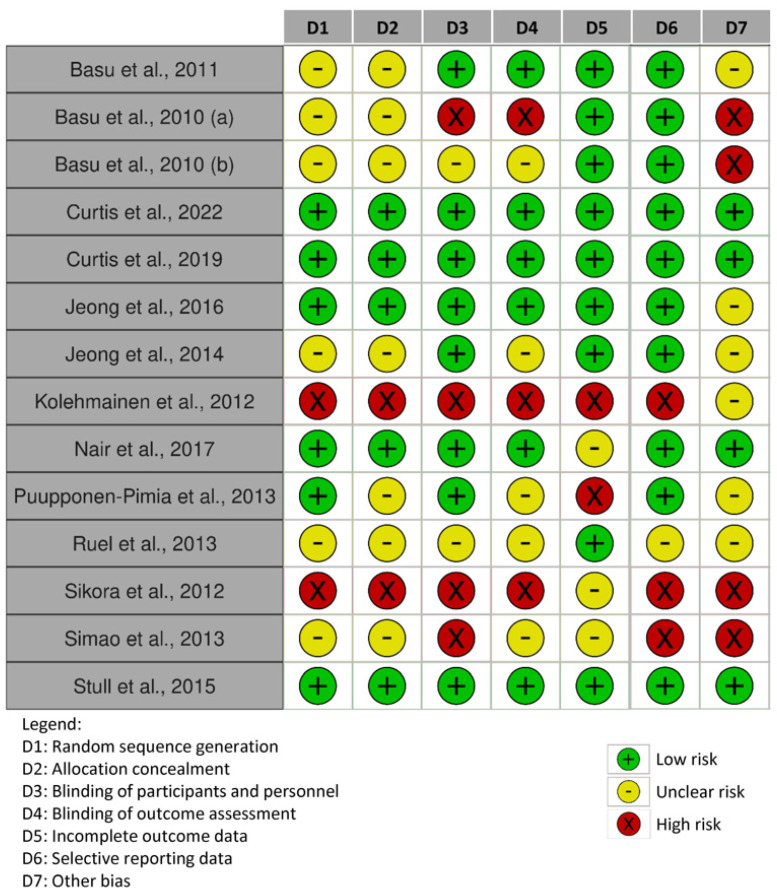
Risk of bias for each domain evaluated within the studies [5,32,33,35,36,37,38,39,40,41,42,46,47].

**Figure 3:**
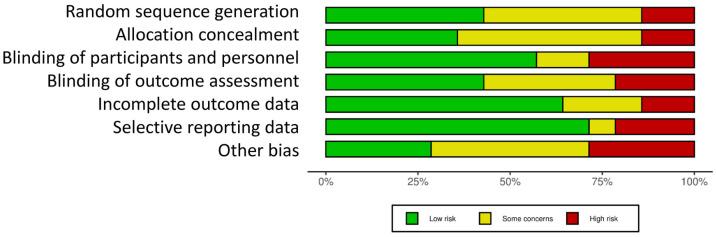
Risk of bias for each domain reported as a percentage across all the included studies combined.

The results of the risk of bias carried out within individual studies and across the studies are reported in Figure 2 and Figure 3, respectively. The risk of bias related to 14 out of 17 studies is reported here. Overall, the results from the RCTs assessment showed that the blinding of participants and personnel and other bias represented the highest risks of bias, followed by blinding of outcome assessment and selective reporting data (Figure 3). The last three studies were analyzed using the risk of bias in non-randomized studies of intervention scale (Supplementary Figure S1), where the major risks of bias were: bias in measurement classification of interventions (100% risk) followed by bias due to confounding and bias in the selection of participants into the study (at about 70% risk) (Supplementary Figure S2).

### 3.4. Main Findings

Most of the trials (six out of seventeen) reported a decrease in triglycerides (TGs) and an increase in high-density lipoprotein cholesterol (HDL-C), which are two components of MetS. Additionally, a positive effect was found on low-density lipoprotein cholesterol (LDL-C) as well, a second marker of T2D, negatively associated with health outcomes (Table 1). This effect is berry dependent, with chokeberry and blueberry the most effective. Conflicting results were observed for the other MetS markers.

Positive findings were documented for markers related to inflammation (Supplementary Table S1). Six out of seventeen studies showed an improvement in at least one or more cytokines. The main effects were related to interleukin-6 (IL-6) and tumor necrosis factor-alpha (TNF-α). A trend towards a positive effect has also been observed for markers of oxidative stress, while little evidence is available regarding markers of vascular health (Supplementary Table S1). A summary of the findings obtained within the berries is below.

#### 3.4.1. Effect of Blueberry (BB)

The effect of BB intervention in subjects with MetS was evaluated in six studies: two studies investigated the post-prandial effect of BB [32,34], while four studies studied the medium-long-term effect [5,33,35,36].

In a parallel, double-blind, randomized, controlled trial, Curtis and colleagues [32] investigated the effect of a freeze-dried BB (26 g equivalent to 150 g of fresh highbush BB) consumed together with an energy-dense food (900 kcal; 500 g milkshake), on the postprandial cardiometabolic response in a group of 45 subjects with MetS. The BB contained 364 mg ACNs and 879 mg phenolics, while the placebo was an isocaloric and carbohydrate-matched purple-colored powder (0 mg ACNs or phenolics) of similar appearance and taste to the freeze-dried BB. Results were adjusted for the use of statins and antihypertensive drugs. The intake of BB + energy-dense food reduced the post-prandial glucose and insulin response and total cholesterol (TC) while increasing HDL-C, Apolipoprotein AI (APO-A1), extra-large, high-density lipoprotein particle number (XL-HDL-P-n), and large, high-density lipoprotein particle number (L-HDL-P-n). Conversely, no effect emerged for blood pressure, flow-mediated dilation (FMD), pulse wave velocity (PWV), and augmentation index (AIX). In a controlled, crossover study, Sobolev et al. [34] evaluated the effect of a postprandial high fat/high glycemic load meal (control treatment) versus a high fat/high glycemic load meal enriched by BB (150 g, test treatment) on metabolomic response in a group of five patients with overweight and/or obesity. The composition of macronutrients and energy between meals (with or without BB) was comparable (energy from lipids > 30% of meal total energy, glycemic load >20 according to the University of Sydney classification system). No information on polyphenol content was available. The authors observed an increase in the expression of transforming growth factor- β (TGF- β) and a reduction in interleukin-6 (IL-6) after 2–4 h from the intake of a high fat and high glycemic load meal + BB. In addition, a decrease in indicators of T2D (i.e., methylamines, acetoacetate, acetone, and succinate) was documented. Furthermore, an accumulation of gut microbial dehydrogenation of proanthocyanidins and procyanidins (p-hydroxyphenyl-acetic acid and 3-(3′-hydroxyphenyl)-3-hydroxypropionic acid) was detected in the urine, suggesting a contribution of the gut microbiota in the metabolization of BB polyphenols. Conversely, no effect on IL-1 β, TNF-α, IL-10, and IL-4 mRNAs gene expression was reported.

The medium-long-term effect of BB was investigated in five studies. In a six-month, double-blind, parallel-arm, randomized, placebo-controlled trial, Curtis and coworkers [33] tested the effect of 1/2-cup (13 g powder, equivalent to 75 g raw highbush BB) or 1-cup (26 g powder, equivalent to 150 g raw BB) of a freeze-dried BB powder versus a placebo, on cardiometabolic and vascular markers in a group of 115 subjects. Participants were instructed to consume BB based on the dietary instructions provided (eight standardized recipes). The 1-cup and 1/2-cup of BB contained ACNs (364 mg and 182 mg, respectively) and phenolics (879 mg and 439 mg, respectively). The placebo was a powder matching the energy and carbohydrate content of BB. Results were adjusted for the use of statins and antihypertensive drugs. While the interventions determined a significant increase in triglycerides (TG) levels following both BB portions compared to placebo, no effect was reported for the other markers related to lipid and glucose profile and blood pressure. In merit to vascular function, the authors observed a significant increase in FMD and cyclic guanosine monophosphate (cGMP) while documenting a reduction in AIx following the consumption of 1/2 cup BB, but not 1 cup.

In a six-week, randomized, double-blind, placebo-controlled trial, Nair et al. [5] evaluated the potential role of two highbush BB species in the modulation of the immune response and inflammatory and oxidative stress response in subjects with MetS. The participants consumed twice daily ∼356 mL yogurt and a skim-milk-based smoothie prepared by adding 22.5 g of freeze-dried BB powder (a total of 45 g per day) or a placebo (an identical smoothie without the BB powder). The intervention reduced oxidative stress by decreasing free radical levels both in the whole blood as well as isolated monocytes. In addition, a decrease in circulating inflammatory markers such as TNF-α, Toll-like receptor 4 (TLR4), IL-6, and granulocyte-macrophage colony-stimulating factor (GM-CSF) was observed. Conversely, a significant increase in the immune system, in terms of myeloid dendritic cells, was reported. In a previous six-week, randomized, double-blind, placebo-controlled trial, Stull et al. [35] investigated the effect of a BB smoothie on blood pressure, endothelial function, and insulin sensitivity in a group of obese subjects with MetS. The authors documented an improvement in peripheral arterial tone (a marker of vascular function) following BB compared to placebo, while no changes were reported for the rest of the parameters considered (e.g., body weight and body composition, blood pressure, glucose and insulin, and lipid concentrations).

Finally, in an eight-week, single-blind, controlled study, Basu et al. [36] examined the impact of BB on lipid peroxidation and inflammation in a group of obese men and women with MetS. Subjects consumed a freeze-dried BB beverage prepared by suspending 50 of powder (corresponding to 350 g fresh BB) in 480 mL of water or an equivalent amount of liquids as control treatment. One cup (240 mL) was consumed in the morning, while the second cup (240 mL) was in the evening. BB provided 1624 mg of phenolics and 724 mg of ACNs. Overall, the consumption of BB improved systolic and diastolic blood pressure, plasma-oxidized LDLs (ox-LDLs), malondialdehyde (MDA), and 4-hydroxynonenal (HNE) levels. Blood pressure outcomes remained significantly different also when data were analyzed without participants on stable antihypertensive medications. No effect was recorded on anthropometric, lipid profile (TC and TG levels), myeloperoxidase (MPO), adiponectin, inflammatory markers (C reactive protein -CRP-, adiponectin and IL-6), and adhesion molecules (ICAM-1 and VCAM-1).

#### 3.4.2. Effect of Bilberry (BiB)

The effect of BiB in subjects with MetS was evaluated in one medium-long-term intervention. Specifically, Kolehmainen et al. [37] performed an eight-week, randomized, controlled trial with BiB in a group of 15 subjects with overweight or obesity and MetS. The bilberry (400 g/day) comprised 200 g of BiB purée (providing 1048 mg ACNs and 24 mg flavonols) and 40 g of dried BiB (eq. 200 g of fresh fruit), providing 332 mg ACNs and 12 mg flavonols. The control diet (n = 12 subjects) was composed of the habitual diet of the participants. The results documented a decrease in high-sensitive CRP (hs-CRP), IL-6, IL-12, lipopolysaccharides (LPS), and inflammation score following BiB intervention. These changes were found to be significantly correlated with differentially expressed transcripts related to the TLR signaling pathway, as well as monocyte or macrophage-associated genes. No significant findings were detected for body weight and body composition, glucose, and lipid metabolism.

#### 3.4.3. Effect of Cranberry (CB)

The role of CB on MetS was investigated in three medium-long-term interventions. Ruel et al. [39] analyzed the effect of a four-week, double-blind, placebo-controlled crossover intervention with CB on arterial stiffness in subjects with abdominal obesity with MetS. For the study, volunteers consumed a low-calorie (27%) CB juice or a placebo drink. Specifically, subjects consumed two boxes of juice in the morning and two in the evening (125 mL each). The CB serving provided about 400 mg phenolics, 296 mg proanthocyanidins, and 21 mg ACNs. The placebo drink was comparable in terms of energy, sugars, and ascorbic acid to the CB juice. The intervention with CB decreased global endothelial function and arterial stiffness (pre-to-post intervention), but this effect was not significant compared to the post-placebo. No effect on blood pressure, cell adhesion molecules (ICAM, VCAM, and E-selectin), as well as ox-LDLs, was documented.

In an eight-week, non-randomized, controlled, parallel intervention, Simao and coworkers [38] assessed the effect of a CB juice on metabolic and inflammatory biomarkers in MetS patients. To this aim, one group of subjects consumed 700 mL/day of a reduced-energy CB juice (containing 230 mg proanthocyanidins and 360 mg phenolics), while the second group continued with their habitual diet. The intervention with CB led to increased serum adiponectin and folic acid levels while reducing the levels of serum homocysteine, lipid, and protein oxidation compared to the control group. After adjustment for anti-hypertensive drugs, statistical analyses reported a non-significant difference between groups for these patients. Conversely, anthropometric, CRP, TNF-α, IL-1, and IL-6 serum levels were not affected following the intervention.

Finally, in an eight-week, placebo-controlled, randomized intervention, Basu and colleagues [40] evaluated the capacity of a low-energy CB juice to decrease lipid peroxidation, inflammation, and dyslipidemia, in a group of obese women with MetS. Subjects consumed 480 mL/day of CB juice or the same amount as a placebo. The cranberry juice provided about 450 mg of phenolics, 24 mg of ACNs, and 240 mg of proanthocyanidins. The placebo contained the same quantity of sugars and ascorbic acid of CB juice, but no polyphenols were present. The treatment with cranberry reduced circulating levels of ox-LDLs, MDA, and HNE, while also being able to increase total plasma antioxidant capacity. No effect was reported for blood pressure and markers related to lipid and glucose profile, as well as inflammation such as CRP and IL-6.

#### 3.4.4. Effect of Raspberry (RB)

Two medium-long-term interventions evaluated the effect of RB on MetS. In a placebo-controlled, parallel intervention, 51 patients with MetS were randomized into two groups; one group received four capsules/day of black RB powder, while the second group was the placebo group [41]. The black RB capsule contained 187.5 mg of powder (750 mg per day) and other ingredients (magnesium stearate, silica, and isomaltose, while the placebo capsules had the same appearance but contained mainly isomaltose followed by the other ingredients. The black RB powder provided mainly catechin (371 µg) and proanthocyanidin (195 µg) followed by other polyphenols (i.e., anthocyanins and epicatechins), but in minor concentrations. The intervention reduced serum TNF-α, IL-6, ICAM, and Aix, while it improved serum levels of adiponectin and circulating endothelial progenitor cells. No effect on CRP and sVCAM-1 was observed.

In a previous study [42], the same authors performed a 12-week, randomized, double-blind, placebo-controlled intervention in a group of 77 MetS patients. A group received 750 mg/day of black RB (equivalent to four capsules/day), while another group consumed the same number of placebo capsules (capsules with the same appearance but containing cellulose, isomaltose, and corn powder). No data on polyphenol content was reported. The intake of RB capsules led to decreased serum TC and TC/HDL-C ratio, while no effect was observed for the serum lipid profile. Regarding inflammation and vascular function, IL-6 and TNF-α significantly decreased while the anti-inflammatory adiponectin and the values of brachial artery FMD significantly increased compared to placebo. Conversely, CRP, ICAM-1, and VCAM-1 were unaffected.

#### 3.4.5. Effect of Strawberry (StrB)

Two studies evaluated the effects of StrB on MetS. Recently, Basu et al. [51] performed a 14-week, randomized, and controlled, crossover study, in which the effect of two different dosages of a StrB drink was evaluated on the antioxidant status and biomarkers of endothelial function in adults with MetS. For the study, participants were allocated to one of the following three groups: placebo group, low-dose group (13 g/day StrB powder per day), and high-dose group (32 g/day StrB powder per day). Each treatment was four weeks long and separated by a one-week washout period. Total polyphenol content was 400 and 960 mg per one and two-and-half servings, respectively, while total ACN content was 38 and 92 mg (daily dose), respectively. Conversely, the placebo provided the same amount of carbohydrates and energy of StrB, while it did not provide bioactive compounds. Both treatments (low and high StrB dose) significantly increased serum antioxidant capacity and superoxide dismutase activity while decreasing lipid peroxidation, VCAM-1, and TNF-α. No effect on catalase, glutathione peroxidase, glutathione reductase, nitrite, MDA, as well as soluble E-selectin, P-selectin, ICAM-1, IL-6, and IL-1ß, was reported.

In 2010, the same authors [46] performed an eight-week, randomized, controlled, parallel study in which the effect of an StrB drink versus a placebo drink was tested on blood pressure, glucose and lipid metabolism, and circulating adhesion molecules in obese subjects with MetS. To this aim, four cups of water a day (as control) or StrB (two cups of StrB beverage + two cups of water a day) were provided. Each cup contained 25 g of freeze-dried StrB powder equivalent to 500 g (at about three cups) of fresh product. The StrB drink provided about 2000 mg of total phenolics and 154 mg of total ACNs. The authors showed a reduction in TC and LDL-C, small LDL particles, and VCAM-1, while for the rest of the markers, the effect was not significant.

#### 3.4.6. Effect of Chokeberry (ChB)

Three studies investigated the effect of ChB in subjects with MetS. Tasic and coworkers [43] examined the effect of the supplementation with a standardized *Aronia L. Melanocarpa* Extract on several clinical and biochemical parameters in patients with confirmed MetS. The study was a four-week prospective, open-label, non-randomized, non-controlled clinical study during which subjects consumed, in the form of a solution, a dose of 30 mL per day of extract containing 431 mg polyphenols and 120 mg ACN. Overall, TC levels were significantly reduced after two weeks of intervention but only in the group of females with T2D compared to their baseline values, while the LDL-C levels decreased only in the group of non-diabetic females. TG significantly diminished following four weeks of supplementation only in the diabetic groups, compared to the baseline. Additionally, no effect was observed for anthropometric, hematological, or inflammatory parameters.

Sikora et al. [44] performed an eight-week, non-randomized, non-controlled trial aiming to evaluate the impact of the ChB supplementation on platelet aggregation, clot formation, and lysis in patients with MetS. Subjects were on a low-fat diet and received 300 mg of ChB extract per day during the entire study period. The control group was composed of non-treated healthy people. The ChB extract provided about 60 mg of total polyphenols, including a minimum of 20 mg of ACN (mainly 3-O-cyanidin-galactoside and arabinoside). The administration of ChB extract decreased levels of serum TC, LDL-C, and TG, as well as markers related to platelet aggregation and coagulation, with no changes occurring for BMI, WS, and HDL-C.

Finally, in 2010, the same authors carried out an eight-week, non-randomized, non-controlled trial in which the effect of the ChB supplementation on blood pressure, serum endothelin, lipid, and oxidative stress markers was evaluated in patients with MetS [45]. For more details about the study design and the Aronia extract composition, please see Sikora et al. [44]. The intervention reduced blood pressure, endothelin-1, improved lipid profile, and oxidative status, while no effect was observed for BMI, WC, and CRP.

#### 3.4.7. Effect of Berry Mix

One study investigated the role of berry mix on MetS. Puupponen-Pimiä et al. [47] performed a 12-week randomized, controlled, parallel intervention that investigated the effect of a mix of berries on lipid profile, gut microbiota composition, and urolithin production in a group of subjects with MetS. Subjects consumed 300 g/day of fresh berries (100 g of strawberry purée, 100 g of frozen raspberries, and 100 g of frozen cloudberries) or a control diet constituted by their habitual diet restricted in berries consumption. The berries provided about 789 mg ellagitannins, 70.7 mg ACNs, and 4.1 mg flavonols. Overall, the results have shown a reduction in serum leptin levels, which disappeared after a post-hoc analysis, while no significant change occurred for anthropometric parameters, blood pressure, lipid profile, as well as for 8-isoprostanes, and total plasma antioxidant capacity. The microbiota composition changed in both the experimental groups (berry vs. control group). In particular, subjects exhibiting a modification in the composition of the gut microbiota were also those who produced urolithins. This result indicated that the berry ellagitannins bioavailability was strictly dependent on the composition of gut microbiota. No effect in the serum lipidomic profile was observed.

## 4. Discussion

The following systematic review aimed to provide an overview of the evidence deriving from human intervention studies testing the effect of berries and their bioactive compounds in the modulation of MetS. We found very few studies characterized by a large heterogeneity in terms of experimental design, length of intervention, doses of berry, and results. The main studies were focused on blueberry, followed by those on cranberry and chokeberry, while very few were focused on bilberry, strawberry, and raspberry. Most of these latter were focused on subjects at risk of MetS and, for these reasons, were not included. Overall, the present systematic review did not show an effect of berry consumption on MetS parameters, apart from an effect on lipid profile.

Blood lipid levels, especially TC, and LDL-C, represent an established predictor of CVD risks [52] and related complications, including MetS. It has been reported that each 1 mmol/L reduction in LDL-C level can be associated with a 23% risk reduction of major vascular events for a statin and a 25% risk decrease for non-statin interventions, including dietary approaches to lower LDL-C. The role of berries on markers of lipid profile has been studied in several trials. Most of them have been shown to decrease TC and LDL-C, TGs, and increase HDL-C [53,54] in subjects with one or more cardiometabolic risk factors. These improvements could be explained by the ability of berry bioactives to increase hepatic synthesis of apolipoprotein A-I and downregulate the fatty acid synthesis. In this review, we found that chokeberry and blueberry were the berries able to improve lipid profile in the context of MetS by reducing TC, LDL-C and TG levels. However, the results were not univocal for all the studies, particularly for those on blueberry. Our observations are in line with a different meta-analysis of RCTs showing controversial results on the capacity of berries to affect lipid markers. Xu and coworkers [22] reported that the habitual intake of ACN-rich berries significantly reduced only TC but not the rest of the lipid markers, while another meta-analysis of 14 RCTs reported no effect of berries and other fruits [55]. More convincing findings were observed when considering the effects of ACNs (the main berry bioactives). For example, the same meta-analysis of RCTs showed that the supplementation with purified ACNs significantly reduced blood LDL-C and TGs, while increasing HDL-C [55].

Hypertension represents another important risk factor for CVD and one of the MetS components. The reduction of the prevalence of pre- and hypertension represents one of the challenges of the World Health Organization’s Global Action Plan for 2013 to 2020 aiming to decrease premature CVD deaths by 2025 [56]. It has been recently reported that for each 5-mmHg reduction in systolic blood pressure, the risk of CV events decreases by 10% [57]. Berries and berry-bioactives play a potentially beneficial effect on blood pressure. We have recently summarized the major mechanisms through which berry-ACNs may directly or indirectly affect blood pressure [58]. The three main mechanisms involved include (1) the increase of endothelial NO synthase (eNOS) expression and activity reflecting a major production of nitric oxide (NO), (2) the protection of NO from oxidative stress, damage and conversion into peroxynitrite, and (3) the reduction in the synthesis of vasoconstrictors such as angiotensin II, endothelin 1, and thromboxanes. In the present review, only three studies (one on blueberry and two on chokeberry) reported an effect on blood pressure in subjects with MetS. Other studies evaluated the role of berries in the modulation of blood pressure, but in healthy subjects, at risk of CVD. For example, a systematic review of 86 dietary intervention studies by Vendrame et al. [59] concluded that there was not sufficient evidence supporting the existence of a direct blood pressure-lowering effect from berries consumption, while stronger evidence emerged for specific types of berries, such as chokeberry, to indirectly affect blood pressure in subjects with hypertension. Another meta-analysis of 10 RCTs reported a significant reduction in SBP (at about 3.68 mmHg) and DBP (at about 1.52 mmHg) following the intervention with berries, in particular cranberry [55]. Conversely, a more recent pooled analysis of six clinical trials on raspberry and four clinical trials on blackcurrant revealed no significant reductions in blood pressure [60].

Impaired glucose metabolism is another comorbid condition of MetS. The European Association for the Study of Diabetes, together with the American Diabetes Association, recommend lowering hemoglobin A1c (HbA1c) to <7.0% to a reduction of the incidence of microvascular disease [61], and recently, a consensus from both scientific societies has been published for the management of hyperglycemia in T2D patients, in which diet plays an important role [62]. Berries and berry polyphenols may affect glucose metabolism, as has emerged in several studies [29,53,63]. The anti-diabetic effects seem to be attributed to their beneficial effects in protecting pancreatic β-cells, affecting glucose digestion, absorption, and uptake, and activating glucose/lipid metabolism pathways. In addition, berry-bioactive may stimulate glucagon-like peptide-1 (GLP-1), a hormone of the incretin system responsible for the improvement of glycaemic control. GLP-1 is involved in the control of the secretion of insulin and glucagon [64,65] and in the food intake-satiety process. Its increment was associated with slowing gastric emptying and a direct effect on the central nervous system, promoting satiety, and contributing to weight loss.

None of the studies included in the present systematic review analyzed the effect of berries on GLP-1, while most of them evaluated glucose and insulin levels following the post-prandial intervention or after a medium-long-term intervention. Only one study reported a beneficial effect of blueberry on post-prandial glucose and insulin response. Our findings seem in line with other studies. A systematic review and meta-analysis of 21 RCTs showed mixed results on the effect of berries on biomarkers of glucose metabolism [29]. Another meta-analysis reported an effect only on HOMA-IR (as a marker of glucose metabolism) following ACN consumption in patients with varying health status [66], while three meta-analyses showed that the consumption of berries [53,67] and polyphenols [68] significantly lowered HbA1c and fasting blood glucose even if these effects were not of clinical relevance.

Being overweight and obese are important risk factors for MetS. Obesity is characterized by abnormal adipose tissue overgrowth, together with impaired glucose and lipid metabolism [69]. In addition, obesity is also associated with low-grade chronic inflammation, oxidative stress, vascular dysfunction, and dysbiosis [70,71,72]. While numerous studies have reported an inverse association between the consumption of fruits and vegetables, in general, and obesity [73], the evidence related to berries remains unclear. Berry-polyphenols may improve obesity by inhibiting lipid absorption, driving thermogenesis, regulating lipid metabolism, and reducing food intake [74]. Furthermore, they could prevent obesity-associated hepatic steatosis, inflammation, and oxidative stress and modulate the composition of gut microbiota [74,75]. Here, we documented only one dietary intervention showing a significant reduction in body weight, WC, and BMI following berry consumption (chokeberry). The rest of the studies failed to affect anthropometric parameters. In the review of Riordan and Solverson [76], the authors found only a potential protective effect of whole berries, products or berry extracts on clinical parameters related to obesity in human studies, while more convincing evidence was obtained for preclinical models in line with other authors [77]. These conflicting findings could be attributed to the short duration of the studies; in fact, most of them were eight weeks long, while it is well-known that at least 12 weeks of intervention is necessary to detect a reduction of clinical relevance in body weight [52]. Conversely, most of the studies included herein have documented an effect of mitigation on other factors directly or indirectly involved in MetS, such as oxidative stress, inflammation, and vascular function. For example, blueberry, bilberry, and cranberry have been reported to reduce plasma ox-LDL, MDA, and HNE levels, as markers of oxidative stress, as well as plasma IL-6 and TNF-α, as markers of inflammation, while studies with raspberries reported an effect mainly on inflammatory markers. Blueberries, strawberries, and chokeberries have shown an effect on vascular function markers such as FMD, AI, and vascular cell adhesion molecules. The mechanisms mediating their protective effects comprise a decrease in nuclear factor-kappaB signaling, a down-regulation of toll-like receptor 4 signaling, a rise in nuclear factor-erythroid-2-related factor 2, and nitric oxide synthase activity [28].

In vitro and in vivo studies reported the protective role of berries and berry bioactives against inflammation, oxidative stress, and vascular dysfunction [78,79,80]. A meta-analysis of RCTs and observational studies documented that higher fruit and vegetable intake, including berries (strawberries, blueberries, and barberries), was correlated with low circulating levels of CRP and TNF-α but not IL-6 [81]. Xu and colleagues [22], in their meta-analysis of RCTs and prospective studies, documented that ACN-rich berries significantly lower blood CRP, while purified ACNs attenuated CRP and TNF-α. A meta-analysis of 23 trials indicated that berries and berry extracts significantly reduced the levels of several oxidative stress markers (e.g., MDA, ox-LDL, and isoprostane) while significantly increasing the level of total antioxidative capacity and the activity of endogenous enzymes such as SOD and GPx [82]. Regarding vascular function, several systematic reviews, and meta-analyses of RCTs documented an improvement in vascular reactivity, such as FMD, and arterial stiffness, such as AI, following berries [55] or ACN-rich berries [83,84,85,86] intervention.

In the context of the quantities of berries administered, the available studies do not show an effect based on the dose or the composition in terms of specific phytochemicals of the berry. As a result, due to insufficient evidence and the high heterogeneity of studies, it is not possible to identify practical recommendations for the consumption of berries. However, the amounts of consumption recorded at the population level, although increasing, are still lower than the doses generally provided in the studies (range 75–500 g of the fresh product, based on the type of berry). In this regard, it would be appropriate to try to encourage higher consumption of berries to promote their potential effect on human health.

## 5. Strengths and Limitations

The main strengths of the present review regard the systematic approach and the use of different databases for the search allowing it to be more inclusive. In addition, the choice to focus only on subjects with diagnosed MetS by excluding studies carried out in different target populations has allowed us to verify the effect of berries on this target population. This standardization reduced the variability and the potential confounding factors influencing the evidence. An additional strength is the evaluation of the risk of bias of the selected studies able to provide information on their methodological quality. Conversely, there are some limitations worth mentioning. First, the inclusion of low-quality studies (non-controlled, non-randomized) could have limited the evidence on the beneficial effects of berries on MetS. Second, the exclusion of the studies performed in subjects at risk of MetS and/or other conditions could have limited the evidence for different markers or for some berries (i.e., strawberry and blackcurrant). Third, the lack of information on the nutritional composition of some berries (in particular, for their bioactives content) makes it difficult to identify the compound and the dose responsible for the beneficial effects. One drawback of the research is that while most studies indicated that participants adhered to a habitual diet or low-fat diet, they did not offer further details that could facilitate a thorough analysis and yield valuable perspectives. A further limitation is the lack of gender comparisons, which is generally an important factor to be evaluated in clinical and pre-clinical studies. Finally, the lack of adjustment of the results for the use of drugs/medications that could contribute to the overall effect is crucial.

## 6. Conclusions

Overall, despite the selection of studies specifically focused on MetS, we observed a large heterogeneity in terms of results. This could be due to the different foods/products tested, amount/dose, content in bioactives, duration of intervention, and low quality of the studies considered. Many of the studies included were focused on blueberries, cranberries, and chokeberries, while very few were carried out with other berries in this specific target population. The main effects were related to lipid profile and inflammation. These results are in line with the findings obtained in a recent meta-analysis on berries and MetS [15]. However, it will be crucial to perform nutrigenetic and nutrigenomic studies aimed at understanding the interplay between an individual’s genetics and their response to berry intervention. Future studies should also assess gender differences for developing effective and tailored dietary recommendations and interventions that promote better health outcomes for both men and women. In addition, studies should consider the contribution of drugs/medications to the overall effect. Also, due to the pivotal role played by oxidative and inflammatory processes in this context, a step forward would be to include in the definition of MetS of markers of oxidative stress and inflammation. Future well-designed human intervention studies should be performed to substantiate the findings already obtained and provide more evidence about the protective role of berries in the context of MetS. The demonstration of such protective effects in this target population could encourage their use in the dietary approaches for the prevention/management of MetS and alternative or adjuvant to the pharmacotherapy for these subjects.

## Figures and Tables

**Table 1:** Population, Intervention, Comparison, Outcome, and Study design (PICOS) criteria for study selection.

PICOS ITEMS	Inclusion Criteria
Population	Subjects that meet at least three out of five components of MetS
Intervention	Berries, berry extract alone or in combination. No medications
Comparison	No restriction
Outcome	MetS parameters and related markers
Study design	No restriction

**Table 2:** A summary of dietary intervention studies relating to berry consumption in MetS subjects.

Berry	Duration	Study Design	Participants/ MetS Classification	Drug Therapy	Intervention	MetS Findings and Related Parameters	Reference and Country
Blueberry	Single dose	Randomized, double-blind, controlled, cross-over	45 subjects (age: 63 ± 7 y; BMI: 31.4 ± 3.1 kg/m^2^) MetS classification: Harmonized	Antihypertensive medication users (38%) or statin users (44%), or a combination	26 g/day freeze-dried BB (equivalent to 1 cup fresh berries; 150 g) + energy-dense food (900 kcal) Highbush BB (scientific name not reported) Composition: 364 mg ACNs, 879 mg phenolics	↓ GLC (at time 180 min) ↑↑ HDL-C =SBP, DBP Related parameters: ↓ insulin (at time 180 min), ↓ TC ↑↑ APO-A1, ↑↑ L-HDLP ↑ XL-HDLP	Curtis et al., 2022 (UK) [32]
	6 months	Randomized, double-blind, placebo-controlled, parallel arms	115 subjects (age: 63 ± 7 y; BMI: 31.2 ± 3.0 kg/m^2^) MetS classification: Harmonized	Antihypertensive medication users (24%) or statin users (38%), or a combination	Group 1: 26 g/day freeze-dried BB (equivalent to 1 cup fresh berries; 150 g) Group 2: 13 g/day freeze-dried BB (equivalent to 1/2 cup fresh berries; 75 g) Highbush BB (scientific name not reported) Composition: Group 1: 879 mg phenolics, 364 mg ACNs Group 2: 439 mg phenolics, 182 mg ACNs	↓ TG =GLC, HDL-C, SBP, DBP Related parameters: =insulin, HbA1c, TC, TC/HDL-C, LDL-C	Curtis et al., 2019 (UK) [33]
	Single dose	Controlled, crossover	5 subjects (age: 26–61 y; BMI: 28–40 kg/m^2^) MetS classification: NCE-ATP III	No use of statins, anti-diabetics or chronic use of nonsteroidal anti-inflammatory drugs	High fat/high glycemic load meal + 150 g BB BB variety and scientific name not reported Composition: N.A.	N.A. (other markers are reported in Supplementary Table S1)	Sobolev et al., 2019 (Italy) [34]
	6 weeks	Randomized, double-blind, placebo-controlled, parallel arms	27 subjects (age: 56.5 ± 2.5 y; BMI: 34.7 ± 1.1 kg/m^2^) MetS classification: WHO	No use of non-prescription drugs	Twice daily, a 12-oz (∼356 mL) yogurt and a skim-milk-based smoothie with 45 g per day of freeze-dried BB powder (equivalent to approximately 2 cups of fresh berries) Highbush BB Tifblue (*Vaccinium ashei*) and Rubel (*Vaccinium corymbosum*) 50/50 Composition: 773.6 mg total phenolics, 290.3 mg ACNs	N.A. (other markers are reported in Supplementary Table S1)	Nair et al., 2017 (USA) [5]
	6 weeks	Randomized, double-blinded, placebo-controlled, parallel arms	23 subjects (age: 55 ± 2 y; BMI: 35.2 ± 0.8 kg/m^2^) MetS classification: WHO	Antihypertensive medication users (95%)	A skim-milk-based smoothie prepared with 45 g BB powder (equivalent to 2 cups of fresh berries) Highbush BB Tifblue (*Vaccinium ashei*) and Rubel (*Vaccinium corymbosum*) 50/50 Composition: 773.6 mg total phenolics, 290.3 mg ACNs	=GLC, TG, HDL-C, SBP, DBP Related parameters: =BW, BMI, % BF, % lean and fat mass, Insulin, TC, LDL-C	Stull et al., 2015 (USA) [35]
	8 weeks	Randomized, single-blind, controlled, parallel arms	48 subjects (age: 50.0 ± 3.0 y; BMI: 37.8 ± 2.3 kg/m^2^) MetS classification: NCE-ATP III	Antihypertensive medication users (20%) and multivitamin users	480 mL/day BB drink (50 g freeze-dried BB corresponding to 350 g fresh berries) Highbush BB Tifblue (*Vaccinium ashei*) and Rubel (*Vaccinium corymbosum*) 50/50 Composition: 1624 mg phenolics, 742 mg ACNs	↓ SBP, DBP =WC, GLC, TG, HDL-C; Related parameters: =BW, BMI, HbA1C, HOMA-IR, TC, LDL-C	Basu et al., 2010 (USA) [36]
Bilberry	8 weeks	Randomized, controlled, parallel arms	27 subjects (age: 53 ± 6 y; BMI: 31.4 ± 4.7 kg/m^2^) MetS classification: NCE-ATP III	No information	200 g of bilberry purée + 40 g dried bilberries (equivalent to 400 g fresh berries) *Vaccinium myrtillus* Composition: 1381 mg ACNs, 36.4 mg flavonol	=WC, TG, HDL-C, SBP, DBP Related parameters: =BW, %BF, TC, LDL-C, Apo-A1, Apo-B, markers of cholesterol synthesis	Kolehmainen et al., 2012 (Finland) [37]
Cranberry	4 weeks	Controlled, parallel arms	56 subjects (median age: 50 y; median BMI: 31 kg/m^2^) MetS classification: NCE-ATP III	Anti-hypertensive medication users	700 mL/day reduced-energy cranberry juice *Vaccinium macrocarpon* Composition: N.A.	=WC Related parameters: =BMI	Simao et al., 2013 (Brazil) [38]
	4 weeks	Double-blind, placebo-controlled, crossover	13 subjects § (age: 42 ± 11 y, BMI: 29.3 ± 2.8 kg/m^2^) MetS classification: NCE-ATP III	No medications known to affect lipid and insulin metabolism or blood pressure	500 mL/day of low-calorie cranberry juice (27% juice) *Vaccinium macrocarpon* Composition: 400 mg total polyphenols, 20.8 mg ACNs	=SBP, DBP	Ruel et al., 2013 (Canada) [39]
	8 weeks	Randomized, double-blind, placebo-controlled, parallel arms	31 subjects (age: 52.0 ± 8.0 y; BMI: 40.0 ± 7.7 kg/m^2^) MetS classification: NCE-ATP III	Antihypertensive medication users (20%) and multivitamin users (25%)	480 mL/day cranberry juice *Vaccinium macrocarpon* Composition: 229.0 mg total phenolics, 12.4 mg total ACNs, 119 mg proanthocyanidins	=GLC, TG, HDL-C, SBP, DBP Related parameters: =TC, LDL-C, VLDL-C	Basu et al., 2011 (USA) [40]
Raspberry	12 weeks	Randomized, double-blind, placebo-controlled, parallel arms	51 subjects (age: 59 ± 10 y, BMI: 25 ± 4 kg/m^2^) MetS classification: Harmonized	Aspirin, beta blocker, ACE inhibitor, calcium blocker, diuretics, statin users	750 mg/day dried unripe black raspberry powder *Rubus occidentalis* Composition: Reported for the fresh product but not for the powder	=SBP, DBP	Jeong et al., 2016 (Korea) [41]
	12 weeks	Controlled, parallel arms	77 subjects (age: 60.0 ± 9.4 y; BMI: 25.7 ± 4.2 kg/m^2^) MetS classification: Harmonized	Aspirin, beta blocker, ACE inhibitor, statin, users	750 mg/day of black raspberry powder in capsules *Rubus occidentalis* Composition: N.A.	N.A. Related parameters: ↓ TC, TC/HDL-C ratio =Apo-A1, Apo-B, Apo-B/Apo-A1 ratio	Jeong et al., 2014 (Korea) [42]
Chokeberry	4 weeks	Non-randomized, non-controlled	143 subjects (age: 50–60 y; BMI: 29.7–34.4 kg/m^2^) MetS classification: AHA/NHLBI	Statin users	30 mL/day Standardized chokeberry extract *Aronia melanocarpa* Composition: 431 mg polyphenols, 120 mg ACNs	↓ WC, GLC, TG, SBP, DBP =HDL-C Related parameters: ↓ BW, TC, LDL-C =BMI	Tasic et al., 2021 (Poland) [43]
	8 weeks	Non-randomized, non-controlled	38 subjects (age: 42–65 y; BMI: 31.1 ± 3.3 kg/m^2^) MetS classification: AHA/NHLBI	No hypolipemic, hypotensive, anticoagulant, antiplatelet, or profibrinolytic drugs	300 mg/day chokeberry extract *Aronia melanocarpa* Composition: 60 mg total polyphenols, 20 mg ACNs	↓ TG =WC, HDL-C Related parameters: ↓ TC, LDL-C =BMI	Sikora et al., 2012 (Poland) [44]
	8 weeks	Non-randomized, non-controlled	25 subjects (age: 42–65 y and BMI: 31.1 ± 3.2 kg/m^2^) MetS classification: AHA/NHLBI	No medication users	300 mg/day chokeberry extract *Aronia melanocarpa* Composition: 3-O-cyanidin-galactoside (64.5%), 3-O-cyanidin-arabinoside (28.9%), 3-O-cyanidin-xyloside (4.2%), and 3-O-cyanidin- glucoside (2.4%)	↓↓↓ TG, SBP ↓↓ DBP ↑↑↑ GLC, HDL-C =WC Related parameters ↓↓↓ TC, LDL-C =BMI	Broncel et al., 2010 (Poland) [45]
Strawberry	8 weeks	Randomized, controlled, parallel arms	27 subjects (age: 47.0 ± 3.0 y; BMI: 37.5 ± 2.15 kg/m^2^) MetS classification: NCE-ATP III	Control group: (24%; antihypertensive medications users) Strawberry group: (0%)	Four cups daily strawberry drink with 25 g/cup of freeze-dried strawberry powder Strawberry: scientific name not reported Composition: 2 g total phenolics, 154 mg total ACNs	=WC, GLC, TG, SBP, DBP Related parameters: ↓ small LDL-C particles =BW, lipoprotein particle concentrations and size	Basu et al., 2010 (USA) [46]
Berry mix	12 weeks	Randomized, controlled, parallel arms	20 subjects (age: 53.0 ± 6.5 year; BMI: 31.8 ± 4.4 kg/m^2^) MetS classification: NCE-ATP III	No information	300 g/day of fresh berries comprising 100 g of strawberry purée, 100 g of frozen raspberries, and 100 g of frozen cloudberries Berries’ scientific name not reported Composition: 789 mg ellagitannins, 70.7 mg ACNs, 4.1 mg flavonols	=WC, HDL-C, SBP, DBP Related parameters: =BMI, TC, LDL-C	Puupponen-Pimiä et al., 2013 (Finland) [47]

§ subjects with MetS. Legend: The arrow ↑ or ↓ denotes significant increase or decrease, respectively. 1 arrow: significantly different at *p* < 0.05; 2 arrows: significantly different at *p* < 0.01; 3 arrows: significantly different at *p* < 0.001; =: no effect; ACNs: anthocyanins; AHA/NHLBI: American Heart Association/National Heart, Lung, and Blood Institute; APO-A1: apolipoprotein A1; APO-B: apolipoprotein B; BB: blueberry; BF: body fat; BMI: body mass index; BW: body weight; DBP: diastolic blood pressure; GLC: glucose; HbA1c, glycated hemoglobin; HDL-C: high-density lipoprotein-cholesterol; HOMA-IR: Insulin resistance index; N.A.: not available; NCE-ATP III: National Cholesterol Education Program—Adult Treatment Panel III; LDL-C: low-density lipoprotein-cholesterol; L-HDLP: large high-density lipoprotein cholesterol particle number; SBP: systolic blood pressure; TC: total cholesterol; TG: triglycerides; XL-HDLP: extra-large high-density lipoprotein cholesterol particle number; VLDL-C: very low-density lipoprotein; WC: waist circumference. WHO: World Health Organization.

## Data Availability

Not applicable.

## Supplementary Materials

The following supporting information can be downloaded at: https://www.mdpi.com/article/10.3390/nu15081906/s1. Table S1: Effect of berry intervention on markers of oxidative stress, inflammation, vascular function markers, and other related parameters in the context of MetS; Figure S1: Risk of bias for each domain assessed within each non-RCT study; Figure S2: Risk of bias for each domain assessed, shown as a percentage across all the non-RCT studies combined.
