# Supplementary material for: Berry Dietary Interventions in Metabolic Syndrome: New Insights
Source: Nutrients. 2023 Apr 14;15(8):1906. doi: 10.3390/nu15081906 (PMC10142833; doi:10.3390/nu15081906)

Figure S1: Risk of bias for each domain assessed within each non-RCT study.

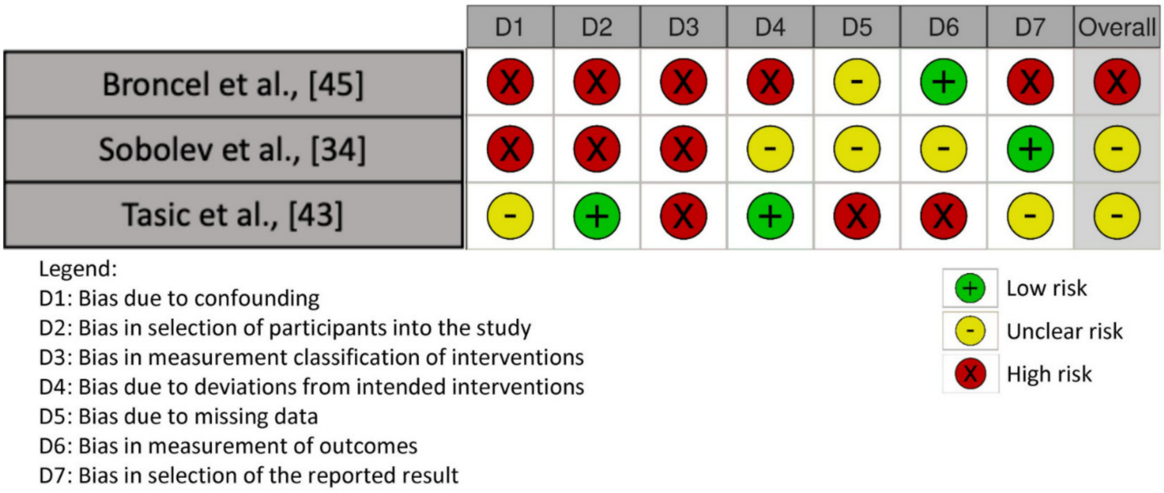

Figure S2: Risk of bias for each domain assessed, shown as a percentage across all the non-RCT studies combined.

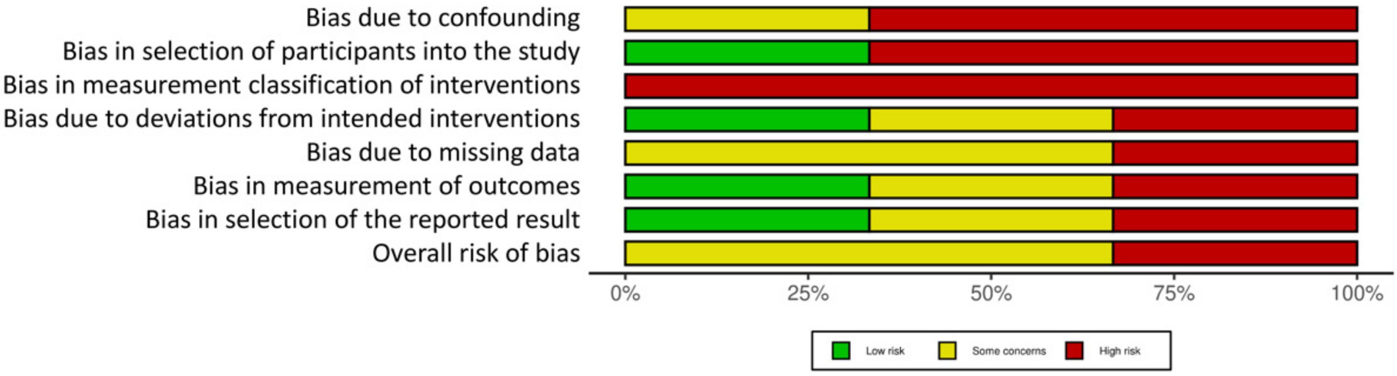

Supplement: Supplementary file 1 [file nutrients-15-01906-s001.zip › supplementary files/Supplementary Figure S1 and S2.pdf]
