# Supplementary material for: Berry Dietary Interventions in Metabolic Syndrome: New Insights
Source: Nutrients. 2023 Apr 14;15(8):1906. doi: 10.3390/nu15081906 (PMC10142833; doi:10.3390/nu15081906)
Supplement: Supplementary file 1 [file nutrients-15-01906-s001.zip › supplementary files/Supplementary Table 1.pdf]

Supplementary Table 1. Effect of berry intervention on markers of oxidative stress, inflammation, vascular function markers, and other related parameters in the context of MetS

Berry	Oxidative Stress markers	Inflammatory/ immune response markers	Vascular function markers	Other markers	Reference and Country
Blueberry					
			= FMD, PWV<br>= AIX		Curtis et al., 2022 (UK) [32]
			↓ AIX@75<br>↑↑ FMD, cGMP		Curtis et al., 2019 (UK) [33]
		↓↓↓ IL-6<br>↑↑ TGF-β<br>= IL-1β, TNF-α, IL-10, IL-4			Sobolev et al., 2019 (Italy) [34]
	↓ free radical levels in the whole blood and isolated monocytes	↓ TNF-α, TLR4, IL-6, GMCSF<br>= IFN-α, IL12p70, FLT3L		↑ myeloid dendritic cells	Nair et al., 2017 (USA) [5]

			↑↑ RHI		Stull et al., 2015 (USA) [35]
	↓↓ ox-LDL, MDA, HNE	= CRP, IL-6	= sICAM-1, sVCAM-1	= adiponectin	Basu et al., 2010 (USA) [36]
Bilberry					
		↓ hs-CRP, IL-12, IS<br>= IL-6		↓ expression of MMD and CCR2 transcripts<br>= adiponectin, leptin	Kolehmainen et al., 2012 (Finland) [37]
Cranberry					
	↓↓ AOPP<br>↓ lipo-peroxidation	= CRP, TNF-α, IL-1, IL-6	↓↓↓ homocysteine	↑↑ adiponectin<br>↑ folic acid	Simao et al.,2013 (Brazil) [38]

	= ox-LDL		= AIX, sICAM-1, sVCAM-1, E-selectin, NOx, MAP	= uric acid, HR	Ruel et al., 2013<br>(Canada) [39]
	↓ ox-LDL, MDA, HNE<br>↑ plasma antioxidant capacity	= CRP, IL-6			Basu et al., 2011<br>(USA) [40]
Raspberry					
		↓ TNF-α, IL-6, CD34/KDR cells<br>↑ CD34/CD133, CD34/CD117 cells<br>= CRP	↓ sICAM-1, radial AIx<br>= sVCAM-1	↑ adiponectin,<br>= HR	Jeong et al., 2016<br>(Korea) [41]
		↓IL-6, TNF-α<br>= CRP	↑ FMD<br>= sICAM, sVCAM	↑ adiponectin	Jeong et al., 2014<br>(Korea) [42]
Chokeberry					

		↓ CRP °		= HR, hematological parameters, hepatic profile	Tasic et al., 2021<br>(Poland) [43]
			↓ platelet aggregation and coagulation		Sikora et al., 2012 (Poland) [44]
	↓↓↓ CAT<br>↓↓ ET-1, TBARS<br>↑↑↑ SOD<br>= GSH-Px	= CRP			Broncel et al., 2010<br>(Poland) [45]
Strawberry					
			↓ sVCAM-1,<br>= sICAM-1		Basu et al., 2010<br>(USA) [46]
Berry mix					
	= 8-isoprostanes			= serum leptin, leptin/BMI and resistin/BMI	Puupponen-Pimiä et al., 2013<br>(Finland) [47]

° In women with MetS only (group: fMetS).

Legend: The arrow ↑ or ↓ denotes significant increase or decrease, respectively. 1 arrow: signifi-cantly different at $p < 0.05$; 2 arrows: significantly different at $p < 0.01$; 3 arrows: significantly different at $p < 0.001$; = : no effect;; AIX: augmentation index; AIX@75: augmentation index normalized to 75 beats per minute; AOPP: advanced oxidation protein

products; BMI: body mass index; CAT: catalase; CCR2: chemokine (C-C motif) receptor 2; cGMP: cyclic guanosine monophosphate; CRP: C-reactive protein; ET-1: endothelin-1; FLT3L: FMS-like tyrosine kinase 3 ligand; FMD: flow mediated dilation; GMCSF: Granulocyte-macrophage colony-stimulating factor; GSH-Px: glutathione peroxidase; HNE: 4-hydroxynonenal; HR: heart rate; hs-CRP: high sensitive C-reactive protein; IS: Inflammation Score (calculated based on the values of hsCRP, IL-6, IL-12, and LPS); IL-1β: interleukin 1β; IL-4: interleukin 4; IL-6: interleukin 6; IL-10: interleukin 10; IL-12: interleukin 12; IL-12p70: interleukin 12 subunit p70; IFN-α: Interferon-alpha; MAP: mean arterial pressure; MDA: Malondialdehyde; MMD: monocyte to macrophage differentiation associated; NOx: total nitrates/nitrites; ox-LDL: oxidized low density lipoprotein; PWV: pulse wave velocity; RHI: reactive hyperemia index; sICAM: soluble intercellular Adhesion Molecule 1; SOD: superoxide dismutase; sVCAM: soluble vascular cell adhesion molecule 1; TBARS: thiobarbituric acid reactive substances; TGF-β: transforming growth factor-β; TLR4: Toll-like receptor 4; TNF-α: tumor necrosis factor-alpha.
